# Association Between Cumulative Exposure to Increased Low-Density Lipoprotein Cholesterol and the Prevalence of Asymptomatic Intracranial Atherosclerotic Stenosis

**DOI:** 10.3389/fneur.2020.555274

**Published:** 2020-11-26

**Authors:** Kaijiang Kang, Yu Wang, Jianwei Wu, Anxin Wang, Jia Zhang, Jie Xu, Yi Ju, Xingquan Zhao

**Affiliations:** ^1^Department of Neurology, Beijing Tiantan Hospital, Capital Medical University, Beijing, China; ^2^China National Clinical Research Center for Neurological Diseases, Beijing, China; ^3^Center of Stroke, Beijing Institute for Brain Disorders, Beijing, China; ^4^Beijing Key Laboratory of Translational Medicine for Cerebrovascular Disease, Beijing, China; ^5^Department of Epidemiology and Health Statistics, School of Public Health, Capital Medical University, Beijing, China; ^6^Beijing Municipal Key Laboratory of Clinical Epidemiology, Beijing, China

**Keywords:** epidemiology, LDL-C, intracranial atherosclerotic stenosis, risk factor, TCD

## Abstract

**Background and Purpose:** Intracranial atherosclerosis has gained increasing attention due to the high risk of recurrent clinical or subclinical ischemic events, while the relationship between low-density lipoprotein cholesterol (LDL-C) measured at a single time point and intracranial atherosclerotic stenosis (ICAS) is inconsistent. This study aims to assess the association between cumulative exposure to increased LDL-C and the prevalence of asymptomatic ICAS.

**Methods:** The Asymptomatic Polyvascular Abnormalities Community study was investigated on the epidemiology of asymptomatic polyvascular abnormalities in Chinese adults. In this study, we included 4,523 participants with LDL-C measured at 3 examinations in 2006, 2008, and 2010. Cumulative exposure to increased LDL-C was calculated as following: LDL-C burden_2006−2008_ = [(LDL-C_2006_-1.8) + (LDL-C_2008_-1.8)]/2 ^*^ time_2006−2008_; LDL-C burden = LDL-C burden_2006−2008_ + LDL-C burden_2008−2010_. Transcranial doppler ultrasonography was performed in 2010 to detecting the ICAS.

**Results:** Of the 4,347 patients, 13.3% (580/4,347) were diagnosed with ICAS. In univariate analysis, the association between LDL-C burden and ICAS prevalence was significant, the odds ratios (95% confidence interval) from the lowest to the highest quartile were 1 (reference), 1.30 (0.99–1.70), 1.32 (1.01–1.73), and 2.14 (1.66–2.75), respectively (*P* < 0.05). After adjustment for potential confounding factors, the same result was reached.

**Conclusions:** Cumulative exposure to increased LDL-C is concentration-dependently associated with increased prevalence of asymptomatic ICAS, especially in those under the age of 65 y or free of hypertension, diabetes mellitus, and hyperlipidemia.

## Introduction

Stroke has now become the most common and disabling disease in China, and ischemic stroke is the primary type (1, 2). Intracranial atherosclerosis has gained increasing attention due to the high risk of recurrent clinical, subclinical ischemic events, and its association with cognitive deficits, especially in non-whites (3, 4). Extracranial atherosclerotic stenosis (ECAS) is more prevalent in the Caucasian population, while intracranial atherosclerotic stenosis (ICAS) is one of the leading causes of ischemic stroke in Asian populations due to the differences in race and lifestyle (1, 2, 5, 6). Some studies have shown that hyperlipidemia, especially increased low-density lipoprotein cholesterol (LDL-C), is an independent risk factor for atherosclerotic stenosis and vulnerable plaque of ECAS (7–9). However, the relationship between the lipid levels measured at a single time point and ICAS is still inconsistent (10–13). Some studies suggested that higher LDL-C levels might be associated with increased risk of ICAS (14). Therefore, this community-based, epidemiological and observational study was conducted to explore the association between cumulative exposure to increased LDL-C and the prevalence of ICAS in the Asymptomatic Polyvascular Abnormalities Community (APAC) study.

## Materials and Methods

### Study Design and Population

**Figure 1:**
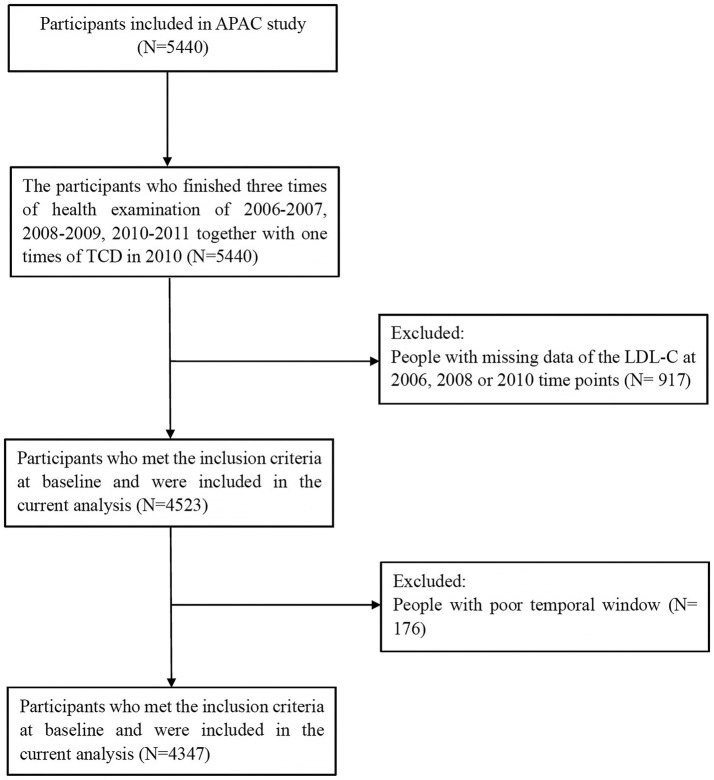
Study flow chart.

The APAC study is a subset of the Kailuan study, investigating the prevalence and associations of asymptomatic multivascular abnormalities in a large industrial population in China (15). A total of 5,440 participants met the inclusion criteria of the APAC study: (1) aged ≥ 40 years; (2) without a history of cardiovascular or cerebrovascular disease. After excluding 917 participants with missing data of LDL-C at 2006, 2008 or 2010 time points, and 176 participants with poor temporal window, 4,347 participants (2,638 men and 1,709 women) were eventually included with full baseline data collected from 2006 to 2010 (Figure 1).

### Measurement of Lipid Levels

Peripheral blood samples were taken from the antecubital vein under fasting conditions and analyzed within 4 h using an auto-analyzer (Hitachi 747; Hitachi, Tokyo, Japan). Lipid parameters, including LDL-C, high-density lipoprotein cholesterol (HDL-C), total cholesterol (TC), and triglyceride (TG), were measured at three examinations in 2006, 2008, and 2010. The specific methods of lipid levels measurement had been described in published articles (8, 9).

### Calculation of LDL-C Burden

Cumulative exposure to increased LDL-C was calculated as the weighted sum of the average LDL-C level:

LDL-C burden_2006−2008_ = [(LDL-C_2006_-1.8) + (LDL-C_2008_-1.8)]/2 ^*^ time_2006−2008_;
LDL-C burden_2008−2010_ = [(LDL-C_2008_-1.8) + (LDL-C_2010_-1.8)]/2 ^*^ time_2008−2010_;
LDL-C burden = LDL-C burden_2006−2008_ + LDL-C burden_2008−2010_.

The cutoff value of LDL-C is set at 1.8 mmol/L (70 mg/dL) according to the SPARCL study, which indicated that achieving an LDL-C level of <1.8 mmol/L was associated with a significant reduction in risk of stroke (16). Participants were classified into four groups (Q1, Q2, Q3, and Q4) according to the interquartile range of LDL-C burden.

### Measurement of Potential Covariates at Baseline

Demographic information, past medical history, and current medications were collected at baseline through a standard questionnaire. Current smoker refers to smoking at least one cigarette per day. Body mass index (BMI) was calculated according to the following equation based on the weight and height: BMI = weight (kg)/square of height (m^2^). Physical activity was categorized as none, seldom, and always. Except for the lipid indicators, systolic and diastolic blood pressure, fasting blood glucose (FBG, hexokinase/glucose-6-phosphate dehydrogenase method) and uric acid (UA, uricase-peroxidase method) were also measured.

### Assessment of Intracranial Atherosclerosis Stenosis

Transcranial doppler ultrasonography (TCD), as a reliable method to diagnose ICAS, was performed by two experienced technicians (blinded to the baseline information of the participants) in 2010 for the detection of ICAS using portable devices (EME Companion, Nicolet, Madison, WI, USA). ICAS was diagnosed according to the peak flow velocity criteria which was published and validated against MR angiography and clinical outcomes (17, 18). Details were described in a previous study. In brief, ICAS was diagnosed if a peak systolic flowing velocity > 140 cm/s for the middle cerebral artery; >120 cm/s for the anterior cerebral artery and internal carotid siphon; and >100 cm/s for the posterior cerebral artery and vertebral-basilar. Also, we considered age, the presence of segmental abnormal velocity, disturbance in echo frequency, or turbulence sound in the diagnosis of ICAS. In this study, the ICAS was diagnosed if stenosis or occlusion was found in one of the studied cranial arteries by TCD, and the participants with poor temporal window were excluded.

### Statistical Analysis

The statistical analysis was performed using a commercial statistical software package (SAS 9.1, Cary, NC, USA). *T*-test or analysis of variance (continuous variables) and chi-squared tests (categorical variables) were used to analyze the differences of baseline characteristics between patients with and without ICAS. Chi-squared tests and logistic regression were used to evaluate the relationship between LDL-C burden levels and ICAS prevalence. Differences of *p* value < 0.05 were considered statistically significant for two-tailed tests.

## Results

### Prevalence of ICAS

Of the 4,347 participants, 580 (13.3%) of them were detected as having asymptomatic ICAS based on the TCD results. The overall distribution and proportion of ICAS in each vessel have been described previously (10, 19).

### Baseline Characteristics

Compared to those without ICAS, the subjects with ICAS were older, with larger BMI, fewer daily activities, higher prevalence of hypertension, DM, hyperlipidemia and current medication in use; and higher levels of systolic or diastolic blood pressure and FBG (*p* < 0.05, Table 1). In addition, when comparing the baseline characteristics according to quartile of LDL-C burden, there were significant differences in age, sex, BMI, physical activity, income status, hypertension, hyperlipidemia, smoking, drinking, systolic and diastolic blood pressure, FBG, and UA.

**Table 1:** Baseline characteristics of the participants with or without ICAS.

**Variables**	**Total patients**	**Non-ICAS**	**ICAS**	***P*-value**
*N*	4,347	3,767	580	
Age, years	53.90 ± 10.92	53.03 ± 10.40	59.54 ± 12.41	<0.01
Male, *n* (%)	2,638 (60.69%)	2,282 (60.58%)	356 (61.38%)	0.713
BMI, kg/m^2^	24.88 ± 3.36	24.84 ± 3.38	25.16 ± 3.20	0.006
Physical activity				<0.01
None	668 (15.37%)	556 (14.76%)	112 (19.31%)	
Seldom	3,104 (71.41%)	2,742 (72.79%)	362 (62.41%)	
Always	575 (13.23%)	469 (12.45%)	106 (18.28%)	
Income status				0.893
<600	1,438 (33.08%)	1,249 (33.16%)	189 (32.59%)	
600–800	2,162 (49.74%)	1,869 (49.62%)	293 (50.52%)	
800–1,000	365 (8.40%)	314 (8.34%)	51 (8.79%)	
>1,000	382 (8.79%)	335 (8.89%)	47 (8.10%)	
Hypertension, *n* (%)	494 (11.36%)	366 (9.72%)	128 (22.07%)	<0.01
Diabetes mellitus, *n* (%)	125 (2.88%)	89 (2.36%)	36 (6.21%)	<0.01
Hyperlipidemia, *n* (%)	307 (7.06%)	243 (6.45%)	64 (11.03%)	<0.01
Current smoker, *n* (%)	1,239 (28.50%)	1,086 (28.83%)	153 (26.38%)	0.224
Current drinker, *n* (%)	1,589 (36.55%)	1,409 (37.40%)	180 (31.03%)	<0.01
Antihypertensive medication, *n* (%)	438 (10.08%)	323 (8.57%)	115 (19.83%)	<0.01
Antidiabetic medication, *n* (%)	106 (2.44%)	74 (1.96%)	32 (5.52%)	<0.01
Lipid-lowering medication, *n* (%)	38 (0.87%)	25 (0.66%)	13 (2.24%)	<0.01
Systolic blood pressure, mmHg	126.43 ± 19.23	124.81 ± 18.39	136.89 ± 21.15	<0.01
Diastolic blood pressure, mmHg	81.11 ± 10.95	80.69 ± 10.80	83.81 ± 11.50	<0.01
Fasting blood glucose, mmol/L	5.41 ± 1.47	5.35 ± 1.37	5.79 ± 2.00	<0.01
Uric acid, μmol/L	287.88 ± 86.02	286.72 ± 85.78	295.43 ± 87.26	0.031

*ICAS, intracranial atherosclerotic stenosis; BMI, body mass index*.

### Correlation Between LDL-C Burden and the Prevalence of Asymptomatic ICAS

There were significant differences in LDL-C levels measured in 2006, 2008, and 2010 between participants with ICAS and those without ICAS (*p* < 0.05) (Table 2). There were also significant differences in LDL-C burden, LDL-C burden_2006−2008_, and LDL-C burden_2008−2010_ between participants with ICAS and those without ICAS (*p* < 0.01) (Table 2). In univariate analysis, the association between LDL-C burden and prevalence of asymptomatic ICAS was significant (*p* < 0.01). Compared with participants in the Q1 group, the Q2, Q3, and Q4 groups had significantly increased prevalence of asymptomatic ICAS with ORs of 1.30 (0.99–1.70), 1.32 (1.01–1.73), and 2.14 (1.66–2.75), respectively (*p* < 0.05). In multivariate analysis, after adjustment for age, gender, smoking, drinking, BMI, hypertension, diabetes mellitus (DM), hyperlipidemia, physical activity, income status, antihypertensive medication, antidiabetic medication, and lipid-lowering medication, similar and significant associations were also observed with ORs of 1 (reference), 1.33 (1.01–1.76), 1.41 (1.07–1.87), and 2.12 (1.63– 2.76) from the lowest to the highest quartile (*p* < 0.05) (Table 3).

**Table 2:** LDL-C and LDL-C burden levels of the participants with or without ICAS.

**Variables**	**Total patients**	**Non-ICAS**	**ICAS**	***P*-value**
*N*	4,347	3,767	580	
LDL-C_2006_, mmol/L	2.31 ± 0.79	2.30 ± 0.77	2.37 ± 0.86	0.04
LDL-C_2008_, mmol/L	2.56 ± 0.96	2.53 ± 0.97	2.74 ± 0.91	<0.01
LDL-C_2010_, mmol/L	2.60 ± 0.73	2.59 ± 0.72	2.69 ± 0.77	<0.01
LDL-C burden_2006−2008_, (mmol/L)*year	1.32 ± 1.56	1.26 ± 1.52	1.65 ± 1.73	<0.01
LDL-C burden_2008−2010_, (mmol/L)*year	1.47 ± 1.32	1.44 ± 1.33	1.69 ± 1.31	<0.01
LDL-C burden, (mmol/L)*year	2.79 ± 2.68	2.70 ± 2.66	3.34 ± 2.79	<0.01

ICAS, intracranial atherosclerotic stenosis; LDL-C burden_2006−2008_ = [(LDL-C_2006_ – 1.8) + (LDL-C_2008_ – 1.8)]/2 * time_2006−2008_; LDL-C burden_2008−2010_ = [(LDL-C_2008_ – 1.8) + (LDL-C_2010_ – 1.8)]/2 * time_2008−2010_; LDL-C burden = LDL-C burden_2006−2008_ + LDL-C burden_2008−2010_.

**Table 3:** Association between LDL-C burden and the prevalence of asymptomatic ICAS.

	**Q1 (−5.07, 1.07)**	**Q2 (1.08, 2.72)**	**Q3 (2.72, 4.24)**	**Q4 (4.24, 57.32)**	***P* for trend**
Events, *n* (%)	106 (9.76%)	134 (12.33%)	136 (12.51%)	204 (18.77%)	
Univariate analysis, OR (95% CI)	1	1.30 (0.99–1.70)	1.32 (1.01–1.73)	2.14 (1.66–2.75)	<0.01
Model 1, OR (95% CI)	1	1.35 (1.03–1.78)	1.46 (1.11–1.92)	2.17 (1.68–2.81)	<0.01
Model 2, OR (95% CI)	1	1.34 (1.01–1.77)	1.41 (1.07–1.87)	2.12 (1.63–2.76)	<0.01
Model 3, OR (95% CI)	1	1.33 (1.01–1.76)	1.41 (1.07–1.87)	2.12 (1.63–2.76)	<0.01
Model 4, OR (95% CI)	1	1.47 (1.05–2.07)	1.59 (1.13–2.24)	2.47 (1.78–3.43)	<0.01
Model 5, OR (95% CI)	1	1.33 (0.96–1.84)	1.51 (1.09–2.08)	2.29 (1.68–3.12)	<0.01

*OR (95% CI): odds ratio (95% confidence interval)*.

*Model 1: adjusted by age and sex on the basis of univariate analysis*.

*Model 2: adjusted by smoking, drinking, BMI, history of hypertension, diabetes mellitus and hypercholesterolemia, physical activity, income status on the basis of Model 1*.

*Model 3: adjusted by antihypertensive medication, antidiabetic medication, lipid-lowering medication on the basis of Model 2*.

*Model 4: excluding subjects with age ≥ 65 y on the basis of Model 3*.

*Model 5: excluding subjects with a history of hypertensive, diabetes mellitus, hyperlipidemia on the basis of Model 3*.

In the sensitivity analysis, after excluding the subjects with age ≥ 65 y or the subjects with a history of hypertensive, DM, or hyperlipidemia, the association between LDL-C burden and the prevalence of asymptomatic ICAS was still statistically significant (*p* < 0.01), additional information was demonstrated in Table 3.

## Discussion

To the best of our knowledge, this is the first investigation using the LDL-C burden to demonstrate the association between LDL-C levels and the asymptomatic ICAS prevalence. In this large and retrospective cohort study, we disclosed that cumulative exposure to increased LDL-C was concentration-dependently associated with asymptomatic ICAS prevalence, especially in those under the age of 65 years or free of hypertension, DM, and hyperlipidemia.

ICAS had been reported to account for about 30–50% of ischemic stroke or transient ischemic attacks in Asia and often results in poor prognosis (3, 13). Confronting with the situation, there is a considerable need to identify and control risk factors of ICAS. It was suggested that hyperlipidemia and higher LDL-C levels were associated with increased risk of ICAS in previous studies based on TCD, computed tomography angiography (CTA), and high-resolution magnetic resonance angiography (HR-MRA) (14, 20, 21). While, no significant association was found in the Chinese Intracranial Atherosclerosis (CICAS) study (22), and only the highest quintile of LDL-C, measured at a single time point, was associated with the prevalence of ICAS in the APAC study (19). The fact that LDL-C levels fluctuate over time within individuals was ignored by previous studies based on a single time point assessment and likely to generate biased estimates of its association with ICAS. Although the LDL-C level has been the primary target for lipid-lowering treatment in the prevention of cardiovascular and cerebrovascular disease in clinical practice, the role of LDL-C in ICAS remains to be elucidated (3, 23–25). The strengths of this study relate to the large sample size of subjects to find the significant association of sustained and increased LDL-C with an elevated risk of ICAS prevalence. As we know, LDL-C levels will be affected by some factors such as participants' short-term diet and physical activity, so one single time point measurement of LDL-C level could not reflect the participants' long-term actual LDL-C level. Instead, LDL-C burden, calculated as the weighted sum of the average LDL-C levels, is a more reliable indicator of long-term LDL-C levels.

The critical process involved in arteriosclerosis is the retention and accumulation of cholesterol-rich lipoproteins (especially LDL) within the arterial intima at sites of predilection for plaque formation (26–29). When the concentration of LDL-C increases to a certain extent, the probability of intimal retention of LDL and the initiation or progressive development of atherosclerosis increases (27, 29, 30). As a well-established risk factor of atherosclerosis, LDL-C has been actually proved not merely a biomarker of increased risk but a causal factor in the pathophysiology of atherosclerotic cardiovascular disease (26), which has prompted us to further investigate the association of LDL-C with the prevalence of ICAS.

Considering that old age, hypertension, DM, hyperlipidemia are previously documented risk factors for ICAS, we performed further sensitivity analysis (13, 21, 31). After excluding the subjects with age ≥ 65 y or those with a history of hypertensive, DM, or hyperlipidemia, the same result was obtained with higher ORs in concentration-depend manners, which indicated that LDL-C burden was concentration-dependently associated with the prevalence of asymptomatic ICAS, especially in those with age < 65 y and those without a history of hypertension, DM, or hyperlipidemia.

Based on the current research results, LDL-C appears to be associated with asymptomatic ICAS. Although LDL-C has been a primary target for lipid-lowering therapy in the prevention of ischemic stroke, whether there is a causal relationship between LDL-C and asymptomatic ICAS remains unclear. Miao et al. reported that the degree of stenosis in asymptomatic ICAS can be alleviated with intensive lipid-lowering therapy using statin for Chinese patients with asymptomatic ICAS (32). Chung et al. found that high-dose statin treatment can significantly decrease the LDL-C levels and effectively stabilize symptomatic intracranial atherosclerotic plaques as documented by HR-MRI (33). Evidence above suggests that LDL-C may play a role in the formation and progression of ICAS, and maintaining LDL in a normal range may be the right choice for preventing ICAS in clinical practice. Given the high prevalence of ICAS and increased risk of ischemic stroke, more researches on mechanisms and prospective intervention studies are needed to provide insight into their relationship.

There were some limitations in our study. First, some participants with nonatherosclerotic diseases may also be included in the presumed ICAS group. Second, our study was based on a randomly selected subgroup of participants of the Kailuan Study that included employees and retirees of the Kailuan Co. Ltd. and the study population was selected using a stratified random sampling method by age and sex. It may not be representative of the population of the Tangshan area in Hebei province despite the large study sample. Third, ICAS was only determined at a single time point in 2010 and no baseline assessment of ICAS was made in 2006 among the participants. Forth, we just investigated the cumulative effect of blood lipid but not other factors on ICAS. Despite these limitations, this was the first large, cohort study to our knowledge that investigate the correlation between LDL-C burden and asymptomatic ICAS so far.

In conclusion, our data show that cumulative exposure to increased LDL-C is concentration-dependently associated with increased prevalence of asymptomatic ICAS, especially in those with age < 65 y and free of hypertension, DM, and hyperlipidemia.

## Data Availability Statement

The raw data supporting the conclusions of this article will be made available by the authors, without undue reservation.

## Ethics Statement

The studies involving human participants were reviewed and approved by the Ethics Committees of the Kailuan General Hospital and Beijing Tiantan Hospital. The patients/participants provided their written informed consent to participate in this study.

## Author Contributions

KK and YW interpreted the data and drafted the manuscript. YJ and XZ conceived and designed the research. AW acquired and analyzed the data. JW and JZ made a critical revision of the manuscript. All authors revised and agreed to be accountable for the content of this work.

## Conflict of Interest

The authors declare that the research was conducted in the absence of any commercial or financial relationships that could be construed as a potential conflict of interest.

## References

[1] Liu L Wang D Wong KS Wang Y. Stroke and stroke care in China: huge burden, significant workload, and a national priority. Stroke. (2011) 42:3651–4. 10.1161/STROKEAHA.111.635755 22052510

[2] Zhou M Wang H Zeng X Yin P Zhu J Chen W. Mortality, morbidity, and risk factors in China and its provinces, 1990–2017: a systematic analysis for the Global Burden of Disease Study 2017. Lancet. (2019) 394:1145–58. 10.1016/S0140-6736(19)30427-1 31248666 PMC6891889

[3] Qureshi AI Caplan LR. Intracranial atherosclerosis. Lancet. (2014) 383:984–98. 10.1016/S0140-6736(13)61088-0 24007975

[4] Waddy SP Cotsonis G Lynn MJ Frankel MR Chaturvedi S Williams JE. Racial differences in vascular risk factors and outcomes of patients with intracranial atherosclerotic arterial stenosis. Stroke. (2009) 40:719–25. 10.1161/STROKEAHA.108.526624 18832745 PMC3830073

[5] Redon J Olsen MH Cooper RS Zurriaga O Martinez-Beneito MA Laurent S. Stroke mortality and trends from 1990 to 2006 in 39 countries from Europe and Central Asia: implications for control of high blood pressure. Eur Heart J. (2011) 32:1424–31. 10.1093/eurheartj/ehr045 21487117

[6] Mazighi M Labreuche J Gongora-Rivera F Duyckaerts C Hauw JJ Amarenco P. Autopsy prevalence of intracranial atherosclerosis in patients with fatal stroke. Stroke. (2008) 39:1142–7. 10.1161/STROKEAHA.107.496513 18309170

[7] Kim JS Nah HW Park SM Kim SK Cho KH Lee J. Risk factors and stroke mechanisms in atherosclerotic stroke: intracranial compared with extracranial and anterior compared with posterior circulation disease. Stroke. (2012) 43:3313–8. 10.1161/STROKEAHA.112.658500 23160885

[8] Wu J Wang Y Wang A Jia J Wang X Zhao X. Association between non-high-density lipoprotein cholesterol levels and the prevalence of asymptomatic extracranial internal carotid artery stenosis in a Chinese community-based study. Eur J Neurol. (2019) 26:740–6. 10.1111/ene.13882 30561873

[9] Wu J Zhang J Wang A Chen S Wu S Zhao X. Association between non-high-density lipoprotein cholesterol levels and asymptomatic vulnerable carotid atherosclerotic plaques. Eur J Neurol. (2019) 26:1433–8. 10.1111/ene.13973 31002203

[10] Wu J Wang Y Wang A Xie J Zhao X. Association between fasting triglyceride levels and the prevalence of asymptomatic intracranial arterial stenosis in a Chinese community-based study. Sci Rep. (2018) 8:5744. 10.1038/s41598-018-24157-w 29636518 PMC5893624

[11] Li X Wang A Wang J Wu J Wang D Gao X. Association between high-density-lipoprotein-cholesterol levels and the prevalence of asymptomatic intracranial arterial stenosis. Sci Rep. (2017) 7:573. 10.1038/s41598-017-00596-9 28373708 PMC5428728

[12] Kim BJ Hong KS Cho YJ Lee JH Koo JS Park JM. Predictors of symptomatic and asymptomatic intracranial atherosclerosis: what is different and why? J Atheroscler Thromb. (2014) 21:605–17. 10.5551/jat.21063 24573015

[13] Ma YH Leng XY Dong Y Xu W Cao XP Ji X. Risk factors for intracranial atherosclerosis: A systematic review and meta-analysis. Atherosclerosis. (2019) 281:71–7. 10.1016/j.atherosclerosis.2018.12.015 30658194

[14] Suri MF Qiao Y Ma X Guallar E Zhou J Zhang Y. Prevalence of intracranial atherosclerotic stenosis using high-resolution magnetic resonance angiography in the general population: the atherosclerosis risk in communities study. Stroke. (2016) 47:1187–93. 10.1161/STROKEAHA.115.011292 27056984 PMC5319392

[15] Wu S Huang Z Yang X Zhou Y Wang A Chen L. Prevalence of ideal cardiovascular health and its relationship with the 4-year cardiovascular events in a northern Chinese industrial city. Circ Cardiovasc Qual Outcomes. (2012) 5:487–93. 10.1161/CIRCOUTCOMES.111.963694 22787064

[16] Amarenco P Goldstein LB Szarek M Sillesen H Rudolph AE Callahan A. Effects of intense low-density lipoprotein cholesterol reduction in patients with stroke or transient ischemic attack: the stroke prevention by aggressive reduction in cholesterol levels (SPARCL) trial. Stroke. (2007) 38:3198–204. 10.1161/STROKEAHA.107.493106 17962589

[17] Gao S Lam WW Chan YL Liu JY Wong KS. Optimal values of flow velocity on transcranial Doppler in grading middle cerebral artery stenosis in comparison with magnetic resonance angiography. J Neuroimaging. (2002) 12:213–8. 10.1111/j.1552-6569.2002.tb00123.x 12116738

[18] Jaiswal SK Fu-Ling Y Gu L Lico R Changyong F Paula A. Accuracy of transcranial Doppler ultrasound compared with magnetic resonance angiography in the diagnosis of intracranial artery stenosis. J Neurosci Rural Pract. (2019) 10:400–4. 10.1055/s-0039-1696586 31595110 PMC6779567

[19] Wu J Zhang Q Yang H Gao X Zhou Y Wang A. Association between non-high-density-lipoprotein-cholesterol levels and the prevalence of asymptomatic intracranial arterial stenosis. PLoS ONE. (2013) 8:e65229. 10.1371/journal.pone.0065229 23734240 PMC3666970

[20] Homburg PJ Plas GJ Rozie S van der Lugt A Dippel DW. Prevalence and calcification of intracranial arterial stenotic lesions as assessed with multidetector computed tomography angiography. Stroke. (2011) 42:1244–50. 10.1161/STROKEAHA.110.596254 21454818

[21] Wong KS Ng PW Tang A Liu R Yeung V Tomlinson B. Prevalence of asymptomatic intracranial atherosclerosis in high-risk patients. Neurology. (2007) 68:2035–8. 10.1212/01.wnl.0000264427.09191.89 17548555

[22] Qian Y Pu Y Liu L Wang DZ Zhao X Wang C. Low HDL-C level is associated with the development of intracranial artery stenosis: analysis from the Chinese intracranial atherosclerosis (CICAS) study. PLoS ONE. (2013) 8:e64395. 10.1371/journal.pone.0064395 23691210 PMC3656851

[23] Mach F Baigent C Catapano AL Koskinas KC Casula M Badimon L. 2019 ESC/EAS guidelines for the management of dyslipidaemias: lipid modification to reduce cardiovascular risk. Eur Heart J. (2019) 41:111–88. 10.1093/eurheartj/ehz455 31504418

[24] Kernan WN Ovbiagele B Black HR Bravata DM Chimowitz MI Ezekowitz MD. Guidelines for the prevention of stroke in patients with stroke and transient ischemic attack: a guideline for healthcare professionals from the American Heart Association/American Stroke Association. Stroke. (2014) 45:2160–236. 10.1161/STR.0000000000000024 24788967

[25] Gu X Li Y Chen S Yang X Liu F Li Y. Association of lipids with ischemic and hemorrhagic stroke: a prospective cohort study among 267 500 Chinese. Stroke. (2019) 50:3376–84. 10.1161/STROKEAHA.119.026402 31658904

[26] Ference BA Ginsberg HN Graham I Ray KK Packard CJ Bruckert E. Low-density lipoproteins cause atherosclerotic cardiovascular disease. 1. Evidence from genetic, epidemiologic, and clinical studies. A consensus statement from the European Atherosclerosis Society Consensus Panel. Eur Heart J. (2017) 38:2459–72. 10.1093/eurheartj/ehx144 28444290 PMC5837225

[27] Tabas I Williams KJ Boren J. Subendothelial lipoprotein retention as the initiating process in atherosclerosis: update and therapeutic implications. Circulation. (2007) 116:1832–44. 10.1161/CIRCULATIONAHA.106.676890 17938300

[28] Skalen K Gustafsson M Rydberg EK Hulten LM Wiklund O Innerarity TL. Subendothelial retention of atherogenic lipoproteins in early atherosclerosis. Nature. (2002) 417:750–4. 10.1038/nature00804 12066187

[29] Schwenke DC Carew TE. Initiation of atherosclerotic lesions in cholesterol-fed rabbits. II. Selective retention of LDL vs. selective increases in LDL permeability in susceptible sites of arteries. Arteriosclerosis. (1989) 9:908–18. 10.1161/01.ATV.9.6.908 2590068

[30] Goldstein JL Brown MS. A century of cholesterol and coronaries: from plaques to genes to statins. Cell. (2015) 161:161–72. 10.1016/j.cell.2015.01.036 25815993 PMC4525717

[31] Holmstedt CA Turan TN Chimowitz MI. Atherosclerotic intracranial arterial stenosis: risk factors, diagnosis, and treatment. Lancet Neurol. (2013) 12:1106–14. 10.1016/S1474-4422(13)70195-9 24135208 PMC4005874

[32] Miao H Yang Y Wang H Huo L Wang M Zhou Y. Intensive lipid-lowering therapy ameliorates asymptomatic intracranial atherosclerosis. Aging Dis. (2019) 10:258–66. 10.14336/AD.2018.0526 31011477 PMC6457052

[33] Chung JW Cha J Lee MJ Yu IW Park MS Seo WK. Intensive statin treatment in acute ischaemic stroke patients with intracranial atherosclerosis: a high-resolution magnetic resonance imaging study (STAMINA-MRI study). J Neurol Neurosurg Psychiatry. (2019) 91:204–11. 10.1136/jnnp-2019-320893 31371644

